# Artificial intelligence in echocardiography: a position statement from the British Society of Echocardiography

**DOI:** 10.1186/s44156-026-00124-4

**Published:** 2026-07-20

**Authors:** Sadie Bennett, Christopher Wild, Maria F. Paton, Kelly Victor, Sitara Khan, John Kirkham, George Gaye, Claire Peacock, Shaun Robinson, Charlotte Manisty, Denisa Muraru, Paul Leeson, Partho P. Sengupta, Daniel X. Augustine

**Affiliations:** 1 Heart and Lung Clinic, University Hospital of North Midlands NHS Trust, Stoke-on-Trent, UK; 2 Cardiovascular Clinical Research Facility, Division of Cardiovascular Medicine, Radcliffe Department of Medicine, University of Oxford, Oxford, UK; 3 Royal United Hospitals NHS Foundation Trust, Bath, UK; 4 Department of Health, University of Bath, Bath, UK; 5 Leeds Biomedical Research Centre, Leeds Teaching Hospitals NHS Trust, Leeds, UK; 6 Leeds Institute of Cardiovascular and Metabolic Medicine, University of Leeds, Leeds, UK; 7 Cleveland Clinic London, 33 Grosvenor Place, London, UK; 8 Department of Cardiology, Frimley Health NHS Foundation Trust, Camberley, UK; 9 Cardiac Rehabilitation Team, University Hospital of North Midlands NHS Trust, Stoke-on-Trent, UK; 10 Department of Cardiology, Imperial Healthcare NHS Trust, London, UK; 11 Department of Cardiology, Barts Health NHS Trust, London, UK; 12 Department of Cardiology, University College of London Hospitals, NHS, Foundation Trust, London, UK; 13 Department of Cardiology, Istituto Auxologico Italiano, IRCCS, Milan, Italy; 14 Department of Medicine and Surgery, University of Milano-Bicocca, Milan, Italy; 15 Department of Cardiology, John Radcliffe Hospital, Oxford UniversityHospitals, Oxford, UK; 16 Division of Cardiovascular Diseases and Hypertension, Department of Medicine, Rutgers Robert Wood Johnson Medical School, New Brunswick, New Jersey, USA; 17 Staffordshire, UK

**Keywords:** Artificial intelligence, Echocardiography, Position statement

## Abstract

Echocardiography is a foundational imaging modality for assessing cardiac structure and function, with a long history of technological advancement. As artificial intelligence (AI) becomes increasingly embedded across healthcare, its potential to enhance the echocardiography workflow, from image acquisition and analysis to reporting and risk stratification, has expanded rapidly. Research activity in this field has grown substantially, yet clinical adoption remains variable and often limited by practical, technical, and governance challenges. In response, the British Society of Echocardiography has developed this position statement to provide a structured, consensus‑driven evaluation of the opportunities, limitations, and requirements for the safe, equitable, and effective integration of AI into echocardiography services. Although centred on the UK context, the principles outlined may be relevant to other healthcare systems with similar models of echocardiography delivery.

**Graphical Abstract:**

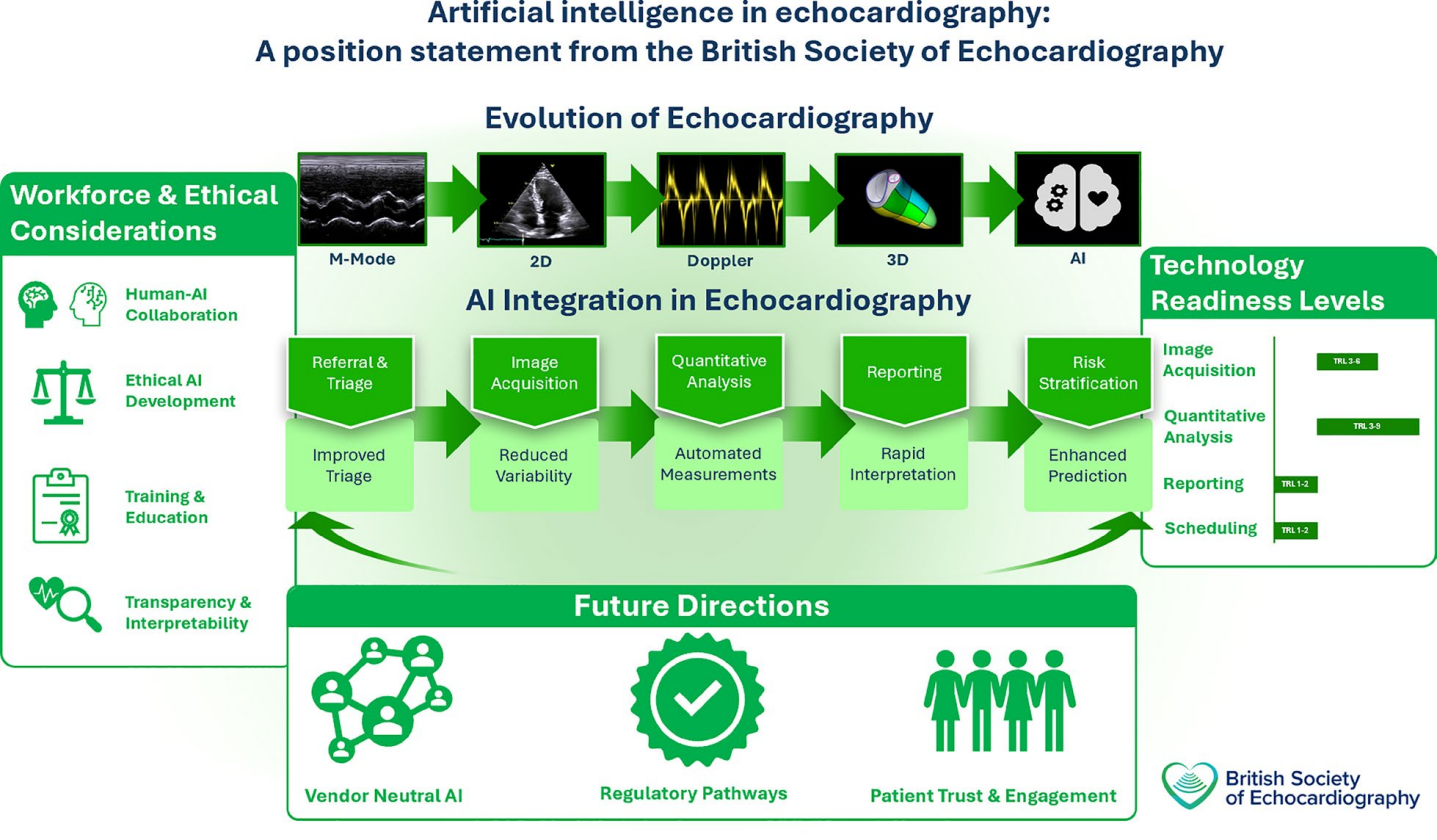

## Introduction

Echocardiography is a cornerstone imaging modality for the assessment of the hearts structure and function, with widespread availability [1] and a long history of technological innovation [2]. As artificial intelligence (AI) becomes increasingly embedded across healthcare, its potential to transform echocardiography, spanning acquisition, analysis, reporting, and risk stratification, has grown rapidly. In recent years, research output in this field has expanded exponentially [3], yet clinical adoption within echocardiography services remains limited and uneven.

Recognising the need for national guidance, the British Society of Echocardiography (BSE) commissioned this position statement to provide a structured, consensusdriven evaluation of the current landscape of AI in echocardiography. The purpose of this document is not to provide a traditional narrative review, but to articulate the BSE’s position on the opportunities, challenges, and requirements for the safe, equitable, and effective integration of AI into echocardiography services. Although the primary focus is the UK context, many of the principles and considerations outlined in this position statement may also be relevant to countries with similar models of echocardiography service delivery.

**Fig. 1:**
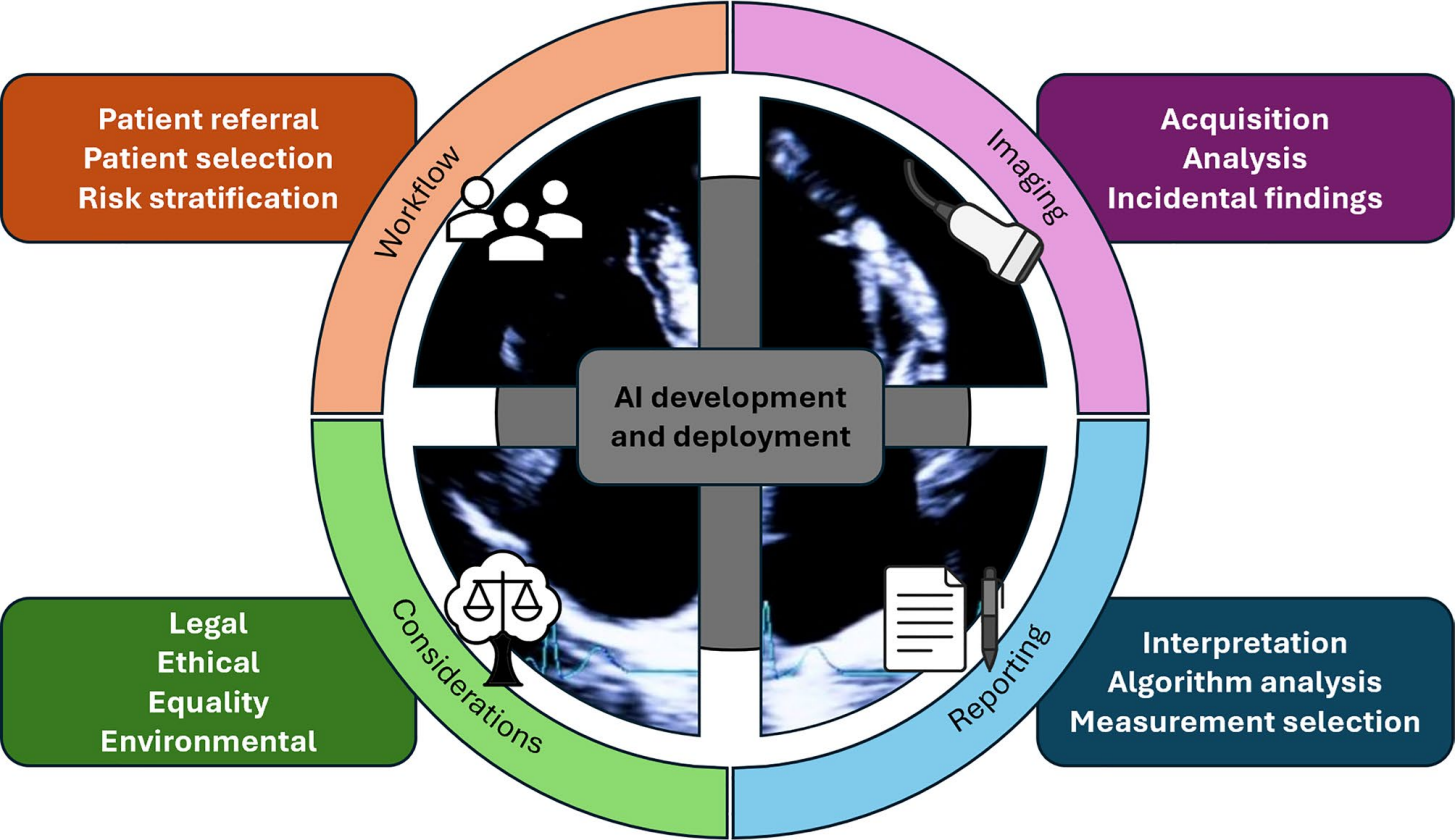
Artificial intelligence application to the echocardiography workflow

To ensure a robust and representative process, the BSE convened a multidisciplinary writing group including cardiologists, cardiac physiologists/clinical scientists, clinical academics, AI researchers, and patient and public representatives. Consensus was achieved through iterative discussion, structured review rounds, and final approval by the BSE Trustees. This document reflects the collective agreement of the group and outlines recommendations for practice, governance, workforce development, and future research developments. This document concentrates on the entire echocardiography workflow (See Fig. 1) and has been informed by a systematic review of the literature (PROSPERO: CRD420251080283). and input from patients and the public. The development of AI within echocardiography is rapidly evolving. As such, the current consensus outline within the position paper will require regular review and where needed, an updated BSE position statement will follow.

## Integration of AI into the clinical echocardiography pathway

**Fig. 2:**
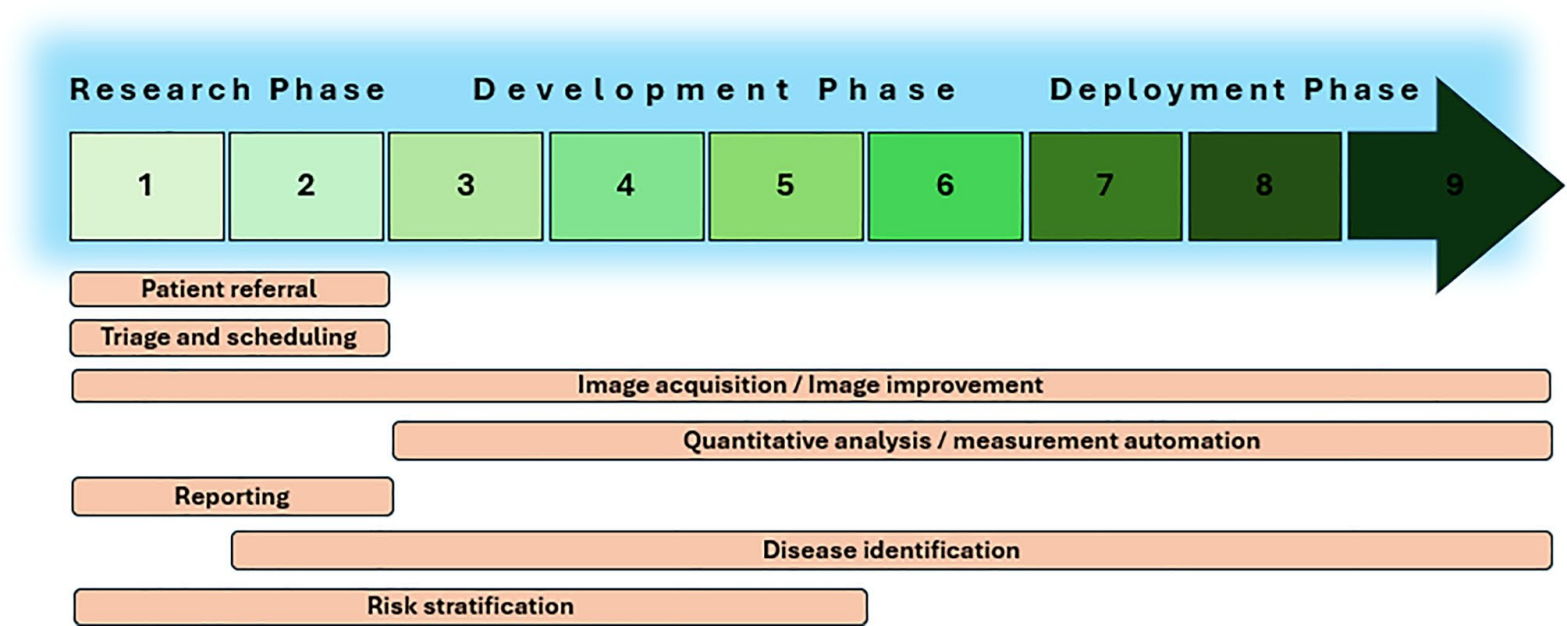
The readiness level of AI models within echocardiograph

The technology readiness level (TRL) scale has been widely used to estimate the maturity and readiness of new technologies that are expected to be deployed within real world settings. This nine-level scale provides a framework that can assess and convey the developmental stage of an AI model, enabling the differentiation of early-stage AI models from those that have gained appropriate regulatory approval, funding approval and have been deployed into real world healthcare settings. The scale has also recently been used within AI and machine learning applications within medical imaging [4–6]. The TRL scale has been adopted in this position statement to clearly distinguish between AI models that are ready for clinical deployment and those that require further evidence (see Fig. 2). To inform the TRL assessments and ensure comprehensive coverage of existing AI echocardiography applications, we conducted a systematic review. Details of this review can be found on PROSPERO (CRD420251080283).

### How can AI help with patient referral, echocardiography triage and appointment scheduling?

A key role for echocardiographers is the interaction with patients, before, during and after their echocardiogram. This will include checking the indications and appropriateness of referral and informing the patient and their family of the findings and next steps. Whilst this interaction is unlikely to be replaced by AI, AI may streamline the initial referral, triage and appointment scheduling. Deep learning models have been shown to support accurate and efficient triage of appropriate referrals [7]. Furthermore, large language models have the potential to support patient referrals and triage by integrating patient information, clinical presentation and societal triage guidelines [8]. This may support echocardiography services in triaging requests more effectively, by enabling the prioritisation of patients based on the clinical need.

The efficient scheduling of echocardiograms has important ramifications in the patient pathway and can help to reduce waiting lists and improve patient access. This scheduling process is often not straightforward and is impacted by the administrative staff availability to coordinate appointments, as well as patients’ availability. By analysing historical data, AI may be able to predict peak times of attendance and demographics of patients who are less likely to attend their appointments at certain times, thus allowing optimal scheduling and service efficiency. At present, there is a lack of AI models focusing on this within echocardiography (TRL 1–2).

### How can AI improve echocardiography image acquisition?

Echocardiography is a specialised image modality requiring highly trained echocardiographers to deliver safe patient care [9]. It involves the acquisition of a comprehensive and standardised imaging dataset within a limited time frame, which can be a challenge for inexperienced echocardiographers. This can be particularly challenging in emergency or community settings, where access to highly trained echocardiographers can be limited [10]. AI models have been shown to improve the acquisition of correct and diagnostic echocardiography images [11–13], which may support the training of novice echocardiographers. This may also aid an early diagnosis and initiation of timely treatment for patients in situations where the availability of experienced echocardiographers is limited. This technology is currently available across a range of echocardiographic vendors (TRL: 9).

AI models are also being developed to improve image quality. Studies have highlighted that deep learning models have the potential to identify and remove noise and artifacts [14]. There is also work on the fusing of multiple 3D echocardiography images with breath holds, enabling the myocardium to be displayed in the same position during different acquisitions [15, 16]. Currently these AI models remain within the research and development phase (TRL: 1–5).

### How can AI models improve quantitative analysis?

Echocardiography is a complex and operator-dependent imaging modality, that requires the acquisition and interpretation of numerous parameters across different acoustic windows [17]. Despite the existence of well-established clinical guidelines, intra- and inter-operator variability remains a significant challenge, often impacting the reliability and reproducibility of echocardiographic measurements [18]. These inconsistencies can affect both diagnostic accuracy and clinical relevance of patients’ longitudinal monitoring using echocardiography. A prime example of this is seen in the echocardiographic assessment of left ventricular ejection fraction, with previous studies demonstrating inter-observer variability of up to ±14% [19]. For patients receiving cardiotoxic chemotherapy, this variability may result in a misjudgement of the left ventricular ejection fraction, potentially causing unnecessary treatment delays [20]. AI models are increasingly demonstrating the ability to standardise measurements and reduce variability, particularly in the quantification of commonly used echocardiographic parameters, including left ventricular ejection fraction [21–25], left ventricular global longitudinal strain [26], valvular heart disease assessment [27–32]. and right ventricular chamber quantification [33]. Whilst guideline-aligned AI quantification is essential for standardised echocardiographic assessment, the emergence of AI-driven interpretative analysis is also a rapidly expanding field, harnessing computational power to detect pathological patterns that may be overlooked by human readers [34]. Automated measurements of the hearts structure and function are now commonplace and available on most up to date echocardiography machines (TRL: 9). The majority of other AI quantification models appear to remain within research/development phase, with few gaining regulatory approval (TRL: 3–9).

### How can AI support echocardiography reporting?

Echocardiography reports should be clear, concise, and effectively communicate key findings to referrers. To support this, guidelines for the assessment of normality and pathology are available [35–44]. However, as echocardiography reports are highly technical, they can be difficult to understand by non-specialists [45]. The use of large language models has the potential to draft automated summaries in a standardised way that answers the clinical question whilst providing guideline-driven findings. Moreover, there may be opportunities for clinical summaries to be automatically converted into patient summaries and translated into his/her native language. Utilising simple, plain language, this could improve the translatability of often complex clinical information to non-specialist referrers and the patients in a timely manner. Additionally, AI models may be able to support with identifying inconsistencies, acting as a ‘sense’ check when different measurements are not consistent from a pathophysiological standpoint, or when measurements and the corresponding text in the conclusions do not match. However, currently these remain within the research/development phase, with none that are specific to echocardiography (TRL: 1–2).

### Where can AI support disease identification on echocardiography?

The use of AI in echocardiography has the potential to enhance the early detection of cardiovascular disease states by analysing features from the images, tabular data, or both in combination, which may enable the uncovering of patterns that are beyond human perception [46]. This is made possible by harnessing vast amounts of diagnostic information from echocardiographic studies within a short time frame.

To date, AI models have shown promising results in several clinical applications. These include the detection of coronary artery disease on stress echocardiography [47], as well as the identification of cardiac amyloidosis [48–50]. AI models have also demonstrated potential in characterising intracardiac masses [6, 9] and in determining the aetiology of left ventricular hypertrophy. In the latter, AI models have been able to accurately and reliably distinguish between athlete’s heart, hypertrophic cardiomyopathy, and hypertensive heart disease [51, 52] Additionally, AI models have shown utility in the assessment of valvular heart disease, including aortic stenosis [27, 31, 53] as well as mitral, tricuspid, and aortic regurgitation [28]. Several AI models have accumulated sufficient evidence to gain regulatory approval, while others remain in earlier stages of development (TRL: 2–9).

### What role does AI play in the risk stratification of patients?

Echocardiography provides important risk stratification information supporting healthcare professionals to make informed decisions on patient care. Traditionally, left ventricular ejection fraction by two-dimensional echocardiography has been the most commonly used prognostic marker, despite its limitations [54]. However, AI models have the potential to identify previously unknown echocardiography markers or imaging phenotypes, that may improve risk stratification and enable the delivery of personalised care. An example of this can be seen in aortic stenosis patients, where an AI model that combines several sources of data (echocardiography, cardiac magnetic resonance, and computed tomography data in addition to blood test results) was able to identify subgroups of aortic stenosis patients who are at higher risk of poor clinical outcomes. This has the potential to improve the timing of aortic valve interventions leading to improved patient outcomes [55]. There is a growing interest in personalised medicine and risk profiling within cardiovascular medicine, and AI is seen as an effective means to achieve this [56, 57]. Currently, echocardiography AI models within this area remain within the research and development phase (TRL: 1–5).

## Key areas of concern and potential solutions

The introduction of AI into echocardiography raises several important workforce considerations. Table 1 outlines the primary concerns associated with integrating AI models into echocardiography practice, alongside suggested mitigating strategies.

**Table 1:** Key areas of concern and potential solutions for the adaptation of AI within echocardiography workflows

Area of concern	Consequences	Potential solutions
*Workforce: Skills*	Potential for the deskilling of the workforce due to both emerging technology becoming the industry standard, and this new technology being poorly understood.	Robust educational foundation in data literacy, AI model interpretation, and quality assurance.
*Workforce: Capacity*	Fatigue/burnout and failure to deliver the perceived service that AI should allow.	A multi-disciplinary approach to understanding the consideration of implementation of an AI model’s ability to expedite service delivery.
*Transparency and equity*	Lack of clarity regarding how AI models are developed, trained, validated, and interpreted. Risk that opaque models exacerbate inequities if performance varies across demographic groups.	Adoption of transparent reporting standards (e.g., model documentation, dataset summaries, interpretability tools). Use of equityfocused evaluation metrics. Inclusion of diverse populations in training and validation datasets. Governance frameworks ensuring explainability and accountability.
*Longitudinal modelling*	Opaque nature of AI training and modelling can result in bias due to poorly trained technology.	Development of methodological guidelines for development and audit to improve reproducibility and overall transparency of AI training and long-term re-training/improvement
*Infrastructure*	Variability in local IT systems, limited storage, lack of interoperability, and challenges integrating AI tools into existing clinical workflows.	Adoption of vendorneutral interoperability standards. Secure computer environments; federated learning frameworks to avoid centralised data pooling. Scalable local or hybrid cloud infrastructure aligned with NHS governance. Careful evaluation of environmental impact when considering large language models or cloudbased generative AI.
*Ethical and legal considerations*	The risk of large-scale data breach resulting in exposure of patient data. Underrepresentation of patient populations leading to bias AI models.	Collaboration between information governance teams, clinical safety officers, cybersecurity, and IT departments. Collaborative approach to the development and sharing of high-quality datasets. Healthcare professions to maintain clinical decision making.
*Environmental concerns*	High energy and water costs involved with running data centres will have a detrimental effect on the environment.	AI models being environmentally friendly by design, and the promotion/implementation of data sharing, renewable energy. The use of these would be ensured by partnering with development companies which adhere to these basic principles.

### Workforce considerations

#### **Risk of deskilling and impact on clinical judgement**

Concerns about deskilling frequently arise in discussions about AI in healthcare. Emerging evidence suggests that exposure to AI models can negatively influence clinician behaviour, potentially contributing to a decline in care standards [58]. When AI outputs are perceived as authoritative or infallible, automation bias [59] may erode critical thinking, leading healthcare professionals to overlook errors or fail to question inaccurate AIgenerated interpretations. If operators are not required to remain actively engaged in controlling or supervising AI algorithms, overreliance can develop, ultimately increasing the likelihood of error compared with scenarios in which clinicians intervene consistently in AIsupported analysis, a phenomenon described as “operator’s dropout.” [60] In parallel, the increasing accessibility of AI tools may foster unwarranted confidence among healthcare professionals with limited subjectmatter expertise, reflecting the Dunning–Kruger effect [61].

#### **Risks introduced by AI-guided acquisition**

AI**-**guided acquisition tools may enable individuals with limited echocardiographic training to obtain diagnostic images. While this capability could expand access in underserved settings, it also introduces the risk that examinations are performed by operators without the foundational knowledge needed to recognise suboptimal windows, artefacts, or clinically significant abnormalities. Without this baseline competence, AIsupported acquisition may inadvertently mask poorquality imaging or delay recognition of important pathology.

#### **Echocardiographer working practices**

Studies have demonstrated that AI assistance can reduce echocardiography time [62], prompting the expectation that such tools could support higher patient throughput. In the context of rising demand for echocardiography services [63] and persistent workforce capacity constraints [64], these findings have fuelled the narrative that AI may enable more efficient workflows and an increase in the number of scheduled echocardiography examinations.

Potential solutions to workforce considerations include human-AI collaboration, quality assurance, retention of healthcare professional oversight, and ongoing professional development. It is widely agreed that AI models within healthcare should be seen as an augmentation tool, with the critical interpretation and final decision-making process remaining with healthcare professionals [65–67]. The integration of AI into clinical practice will demand new competencies beyond traditional training. Specifically, healthcare professionals will need to develop new skills in data literacy, AI model interpretation, and quality assurance to ensure the safe and effective deployment of AI models into patient care [68]. These competencies are essential not only for the accurate use of AI models, but also for recognising their limitations and ensuring clinical accountability.

As demand on echocardiography services continues to rise, the potential for AIenabled reductions in scanning time has been highlighted as a way to lessen the ergonomic burden on echocardiographers. By shortening periods of prolonged static postures, AIsupported workflows may help mitigate the risk of longterm, lifealtering workrelated musculoskeletal disorders [69]. However, these benefits are not universal, as studies also show that in cases with fair or poor image quality, AI tools require more corrections and longer measurement times compared to highquality studies [70]. Moreover, there remains limited realworld evidence from NHS settings to determine whether AI can meaningfully increase patient activity in this setting. Future work should focus on investigating current standard of care whereby many centres already have access to builtin automation within existing echocardiography platforms and continue to operate with 45 to 60 minute appointment timeslots. As such, claims of productivity gains must be interpreted cautiously until robust, contextspecific data is available. If productivity efficiencies are achieved, it is essential to ensure that patient interaction is preserved, and that clinical interpretation and quality assurance of AI-generated results remain central. These safeguards should reinforce shared learning and protect against overreliance on automation.

### Transparency of AI models

The development of accurate, reproducible, and clinically useful AI models in echocardiography depends fundamentally on the quality, completeness, and diversity of the datasets used for training [71]. Models built on incomplete, erroneous, or biased data are at high risk of generating inaccurate predictions and unreliable clinical outputs, limiting their safety and applicability in realworld practice. Alongside this, there is growing demand for greater transparency in AI development [72], including open sharing of training datasets and source code [73, 74] Such openness supports external validation, regulatory scrutiny, and the trust of patients, clinicians, and the wider research community [75]. However, while diverse and representative datasets are essential for developing generalisable AI models, the creation of large, centralised “data lakes” is currently constrained by several practical barriers. These include stringent informationgovernance requirements, variation in local IT infrastructure, limited storage capacity, vendorspecific data formats, and the absence of unified national standards for echocardiography data. As a result, simply advocating for large, pooled datasets is unlikely to be feasible nor sufficient within the current landscape. By emphasising these practical mechanisms, the BSE recognises that the path toward generalisable AI models will depend on collaborative, stepwise, and transparent data**-**sharing approaches, rather than the creation of large, unrestricted national repositories.

A persistent concern in the deployment of AI models, particularly deep learning architectures such as convolutional neural networks, is their “black box” nature, whereby the internal decisionmaking processes are not readily interpretable [76]. While methodological guidelines and reporting frameworks can improve transparency by requiring clear documentation of training data, model architecture, and evaluation procedures [72, 77], these measures alone cannot fully resolve the challenge of explainability.

Transparency also extends to the performance metrics used to assess AI models. Commonly reported metrics that are used in AI echocardiography models have previously been reported [78], However, it is important to note that such metrics can be misleading, particularly in settings with class imbalance when a prevalence of a specific disease is low. In such cases, a model may achieve high accuracy while performing poorly on the minority of positive class. Metrics such as the area under the precision–recall curve may provide a more informative assessment of performance in imbalanced datasets. Clear reporting of dataset composition, including the balance of positive and negative cases, is therefore essential to enable fair interpretation and comparison of model performance.

To ensure safe and trustworthy clinical use, the performance of “black box” AI models must be assessed rigorously in wellpowered, independent external validation cohorts, with comprehensive reporting of dataset characteristics, performance metrics, subgroup analyses, and observed failure modes. Such evaluation is essential to determine whether a model generalises beyond its development environment and to provide clinicians with confidence in its reliability, even when the internal mechanics of the model are not directly interpretable.

### Ethical and regulatory approval considerations

Integrating AI models into cardiovascular imaging requires careful consideration of several ethical principles, including beneficence, nonmaleficence, autonomy, and justice [79]. The datasets used to train AI models are often derived from clinical trials or locally available resources. Yet it is well recognised that certain patient groups, including women, ethnic minorities, and individuals with congenital heart disease or valve repairs/replacements, are often underrepresented. Furthermore, AI models are typically trained, tested and validated on non-UK populations. This lack of representativeness can exacerbate existing health inequalities and contribute to performance bias, whereby AI models underperform for underserved groups [80]. To ensure AI models are equitable and generalisable, both training and validation datasets must reflect the diversity of realworld patients, particularly within UK populations and NHS settings. This includes variation in age, sex, ethnicity, comorbidities, the full spectrum of normal and pathological cardiovascular states, a range of image qualities that support reliable prediction, and differences in equipment across vendors. Such diversity is essential for developing AI tools that are robust, fair, and clinically applicable across varied healthcare settings.

A related ethical consideration concerns the level of information that should be provided to patients when AI models are used within echocardiography workflows. It is not generally necessary to inform each patient individually when AI is used to support routine measurements, provided that clinicians retain full oversight and remain accountable for all measurements entered into the medical record. However, transparency at a service or departmental level is important to maintain trust and uphold patient autonomy. More explicit communication may be required when AI models influence diagnostic interpretation or treatment decisions, where the impact on patient care is more direct and material. Distinguishing between measurementsupport tools and decisionsupport algorithms is therefore essential when determining the appropriate level of patient information.

Before AI models can be deployed and used in healthcare, they must first receive approval under relevant regulatory frameworks. In the UK this must also include approval for funding, typically set by the National Institute of Clinical Excellence. The type of regulatory approval required depends on each country’s jurisdiction and typically involves compliance with data protection laws, medical device regulations, AI-specific governance obligations and other applicable local regulations. In the United States, most AIenabled models Software as a Medical Device is cleared through the Food and Drug Administration (FDA) 510(k) substantial equivalence pathway. This pathway requires evidence that AI models are substantially equivalent to an existing predicate device, but it does not require prospective clinical evidence of safety, effectiveness, or generalisability. As a result, many currently marketed AI tools have limited clinical validation at the time of approval [81]. Comparable regulatory pathways exist in Europe (e.g., Medical Device Regulation) and the UK (e.g., Medicines and Healthcare products Regulatory Agency) [82].

At present, there is no clear consensus on how liability should be assigned when AI contributes to a diagnosis or treatment that results in patient harm [83]. Existing medical liability frameworks are not well suited to the complexities introduced by AI, and there is no comprehensive legal structure defining the responsibilities of developers, suppliers, and endusers [84]. Urgent regulatory action is therefore needed to address the inherent risks of AI, ensure product safety, and maintain minimum safety standards through regular updates. Strengthening legal frameworks governing the use of AI in healthcare is essential to safeguard patient wellbeing.

### Infrastructure considerations

Incorporating echocardiography AI models into healthcare settings requires robust IT infrastructure. This need has been identified as a “bottleneck” in the adoption of AI technologies within healthcare systems, where outdated IT infrastructure limits use and progress [78]. Addressing this issue should be a key priority to ensure equitable access to AI models across all echocardiographic services, regardless of patient demographics or institutional resources. In addition, prior to AI tools being implemented into clinical workflows, there needs to be collaboration between key stakeholders. This includes echocardiographers, data scientists, AI experts, and regulatory bodies. Furthermore, local governance processes will need to be followed, including assessment and approval by local policies regardless of the healthcare setting. This ensures that AI systems meet standards for data protection, cybersecurity, clinical safety and alignment with local digital infrastructure.

An additional consideration is that many echocardiographic AI analysis methods require images to be uploaded to off‑site cloud‑based computing environments for processing, with results subsequently returned to the local system. This workflow introduces further dependencies on secure, high‑bandwidth connectivity, robust cybersecurity safeguards, and clear data‑transfer agreements. These requirements may pose challenges for centres with limited digital capacity or restrictive information‑governance policies and must therefore be factored into local readiness assessments. Importantly, the time required for offsite upload, processing, and return of AIgenerated outputs should also be considered, as this may influence workflow efficiency and the feasibility of integrating such tools into routine clinical practice. Emerging ‘Edge AI’ approaches, which perform AI processing locally on the ultrasound system or within the hospital network, may mitigate some of these challenges, but their availability and performance vary across vendors and device generations.

Prior to implementation, collaboration between key stakeholders, including echocardiographers, data scientists, AI developers, IT teams, and regulatory bodies, is essential. Local governance processes must also be followed, with AI tools assessed and approved in line with institutional policies, regardless of the healthcare setting. This ensures that deployed systems meet standards for data protection, cybersecurity, clinical safety, and compatibility with local digital infrastructure.

### Limitations to the inclusion of artificial intelligence within echocardiography considerations

AI-enabled tools across the echocardiography pathway also have important limitations that must be recognised. For workflow applications such as appointment scheduling and triage, current models may struggle with incomplete or poorquality administrative data, and their outputs can inadvertently reinforce existing inequities if not carefully monitored. AI-guided image acquisition shows promise in supporting lessexperienced operators, yet prospective evidence comparing AI-guided strategies with standard practice remains limited, and errors introduced during acquisition may be difficult to detect downstream. Similarly, while AIbased disease identification has demonstrated encouraging performance in retrospective studies, there is a lack of robust prospective testing in realworld clinical environments, and misclassifications may be harder for clinicians to recognise when model reasoning is opaque. Reporting AI models are also constrained by the absence of consistent definitions, units, and labelling conventions across echocardiography datasets. Although AI may eventually help harmonise the labelling of variables, progress will depend on the adoption of standardised terminology. The BSE is well positioned to support this by promoting consistent labelling standards and encouraging alignment across clinical, research, and vendor communities.

### Future consideration for the application of AI within echocardiography?

There are several areas where the power of AI may be beneficial in improving echocardiography workflows, these include:

1. Automated image stacking and intelligent view organisation
  AI could support more efficient workflows by automatically grouping or “stacking” images according to chamber, valve, or pathologyspecific features. For example, clustering all views relevant to mitral valve assessment would reduce the need to scroll through large image sets and help streamline reporting.
2. Automated comparison of serial studies
  AIdriven tools could enable rapid, standardised comparison of serial measurements across multiple echocardiograms. This may reduce interstudy variability, highlight clinically meaningful changes, and act as a safeguard when subtle differences in image acquisition might otherwise lead to inconsistent interpretation.
3. Integrated audit and qualityimprovement tools
  Embedding AIsupported audit functions that aligned with uptodate echocardiography guidance may help services monitor reporting trends, identify variation in practice, and target educational interventions. Such systems could support continuous quality improvement and strengthen adherence to national standards.
4. Realtime detection of measurement or reporting inconsistencies
  AI could assist clinicians by flagging potential errors or internal inconsistencies before report signoff. For example, highlighting discrepancies between Doppler measurements and narrative conclusions (e.g., a report stating moderate aortic stenosis when haemodynamic parameters indicate severe disease) would support accuracy and patient safety.

## The patient and public perspective of artificial intelligence in echocardiography

The integration of AI into echocardiography requires meaningful engagement with patients and the public to ensure that implementation aligns with their expectations, values, and concerns. To support this, SB/MP conducted a series of patient and public involvement and engagement (PPIE) activities with members of the Stafford and StokeonTrent Heart Support Group (supported by CP)and Leeds Cardiovascular PPIE group. These groups were selected because participants had lived experience of heart disease and were willing to share their views on the use of AI in echocardiography.

Two group discussions were held with the Heart Support Group, followed by onetoone interviews with JK and GG to explore patient and public perspectives in greater depth. A topic guide was used to structure conversations around expectations, concerns, perceived benefits, and priorities for the use of AI in echocardiography. The Leeds Cardiovascular PPIE group discussion was facilitated by a PPIE lead and patient chair, and an open discussion was held following group preference. Ethical approval was not sought, as these activities constituted PPIE rather than research requiring formal review.

**Fig. 3:**
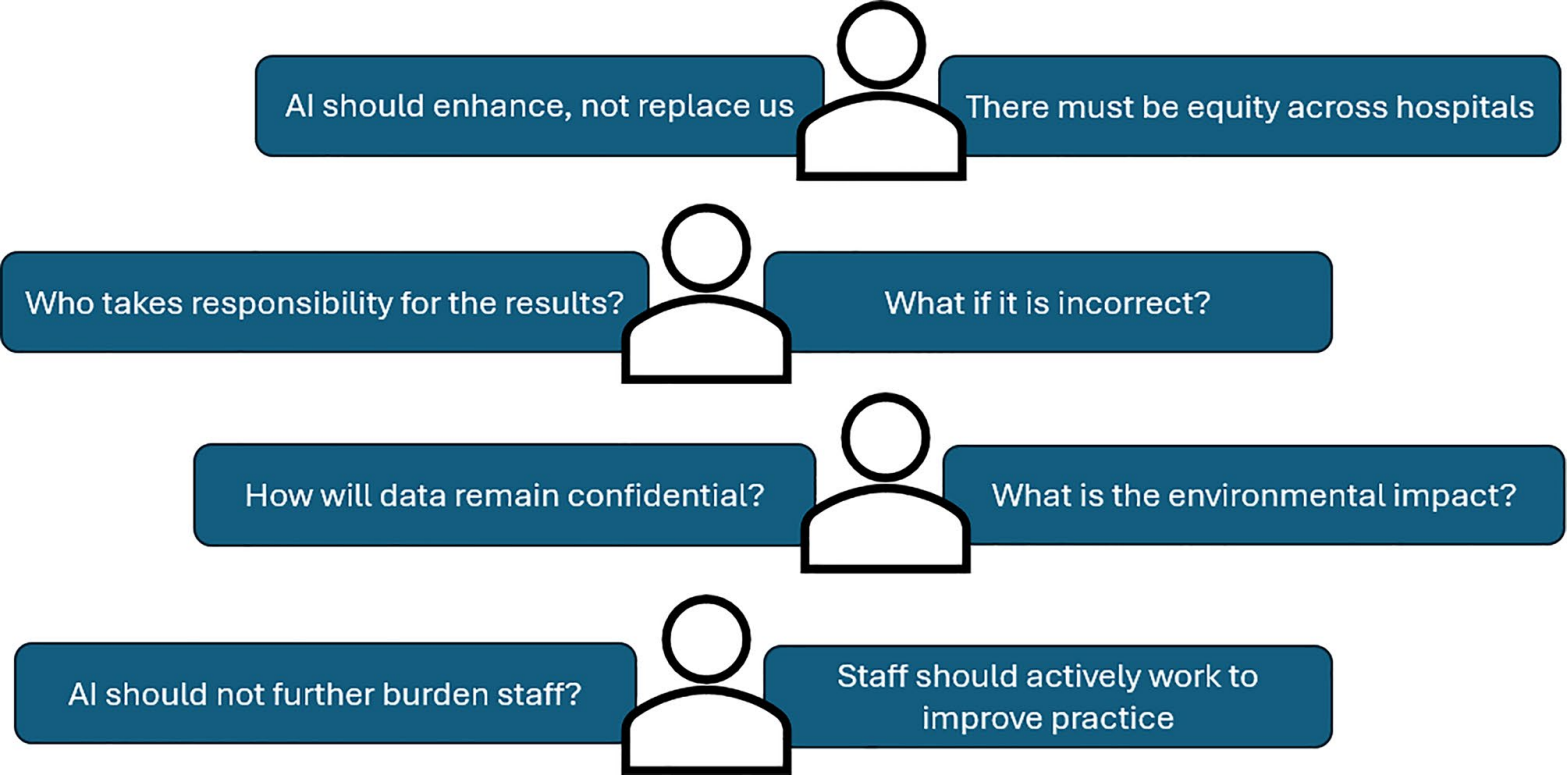
Concerns of patients and the public

The views and perspectives of patients and the public identified several key themes emerged (see Fig. 3). PPIE expressed strong support for the development and use of AI technology, where it could assist healthcare professionals and contribute to improved patient care. There was also a clear message that AI technology should be used to enhance rather than replace healthcare staff to maintain healthcare staff and patient interaction. However, several concerns were raised. A major theme was the importance of ensuring healthcare staff were adequately trained to use AI technology safely and effectively. There was apprehension around whether all healthcare staff would have the necessary skills to interpret and act upon AI-generated results. Concerns were voiced about ensuring healthcare staff maintained the same level of training, knowledge and competency to ensure safe patient care particularly in the instances where the AI technology may be inaccurate or, is unavailable. Additionally, there was an emphasis on healthcare professionals being engaged with research and actively seek opportunities for development to drive practice forwards. Moreover, concerns were expressed about equity of access, particularly whether AI technology would be deployed uniformly across the NHS or remain concentrated in larger, better-resourced hospitals, thus widening health inequalities. PPIE members highlighted practical systemlevel challenges, including the risk of additional burden on existing pathways and the need for reliable transfer of AIgenerated data into electronic health records. Ensuring that these processes are streamlined, interoperable and do not introduce new inefficiencies was seen as essential. PPIE members raised concerns with regards to the use and storage of patient data. Whilst there were general acknowledgment and acceptance that some degree of data sharing was necessary, it was stressed that this must be handled with the highest standards of data security. Ensuring that data remained protected and that the risk of confidentiality breaches are minimised was seen as being essential to maintaining patient trust. Finally, several participants expressed concerns about the environmental impact of AI tools, particularly if there is increased energy usage needed to deliver AI capability. With growing awareness of climate change and sustainability, participants felt it was important that the development and deployment of AI technologies should strive for environmentally responsible approaches wherever possible.

## Future education opportunities

To maximise the value of AI in healthcare and echocardiography, significant investment will be required in education, training frameworks, and governance structures. Recent literature has emphasised the need to develop core knowledge competencies among healthcare professionals to support the safe and effective implementation of AI [85]. These competencies include:

1. **Foundational knowledge of AI**, with a focus on assessing the accuracy, reliability, and validity of AI models.
2. **Data analysis skills**, including data acquisition, cleaning, visualisation, management, and governance.
3. **Understanding of ethical and legal considerations**, particularly in relation to data privacy, accountability, and bias.

**Fig. 4:**
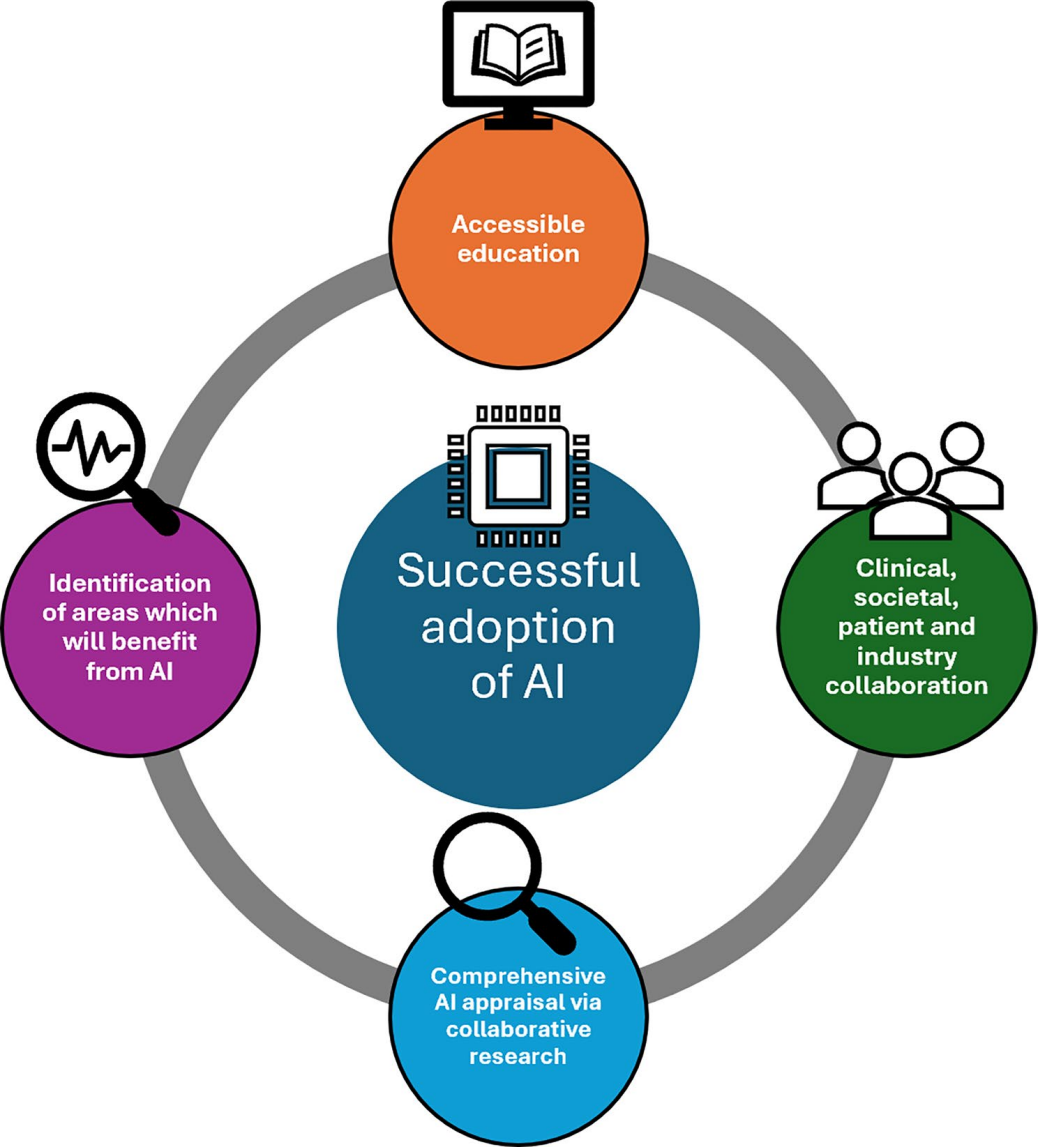
Enabling the safe and effective integration of AI within echocardiography

Building these competencies will equip healthcare professionals with the knowledge, skills, and confidence needed to interpret AI-generated results effectively. In turn, this will support the critical evaluation of AI models, enable clinicians to recognise potential sources of bias, and help prevent the inappropriate use of AI outputs in clinical decisionmaking. Developing these capabilities from the outset is essential to ensure that AI enhances, rather than undermines, patient care [85]. To support the echocardiography workforce in acquiring the necessary competencies and confidence to work safely and effectively with AI, higher education institutions, healthcare training providers, and national professional societies must play a central role (Fig. 4). In line with this, the BSE is committed to ensuring that its members have access to high-quality, AI-specific educational resources. Planned offerings include webinars, e-learning modules, and practical workshops designed to help echocardiographers integrate AI tools confidently into clinical practice. Furthermore, where supported by robust evidence, the incorporation of AI models into future BSE guidance and protocols will be actively considered, helping to embed AI safely, consistently, and transparently into routine echocardiographic workflows.

## Conclusion

Echocardiography remains a central imaging modality for the assessment of cardiac structure and function, and AI now represents the next major phase in its technological evolution. The rapid expansion of AI research demonstrates substantial potential to enhance efficiency, consistency, and diagnostic support across the echocardiography workflow. However, clinical adoption is still limited, and realising the benefits of AI will require careful attention to governance, workforce readiness, data quality, and equitable implementation. To bridge the gap between innovation and routine practice, echocardiography services must learn from other imaging specialties that are further advanced in AI integration, while also responding to the concerns and expectations of patients and the public. Education and upskilling of the echocardiography workforce will be essential to ensure healthcare professionals are confident in evaluating, deploying, and monitoring AI models. As the field continues to evolve, ongoing review of emerging evidence and periodic updates to this position statement will be necessary to ensure that AI is adopted safely, transparently, and in a way that improves patient care across all echocardiography services.

## Data Availability

The data that supports the findings of this manuscript are available from the corresponding author upon reasonable request.
